# Validation of Igbo version of the modified falls efficacy scale among community-dwelling older adults: a validation study

**DOI:** 10.1186/s12955-020-01547-1

**Published:** 2020-09-01

**Authors:** Emmanuel Chiebuka Okoye, Christopher Olusanjo Akosile, Fatai Adesina Maruf, Ifeoma Uchenna Onwuakagba, Sunday Tobias Urama

**Affiliations:** Department of Medical Rehabilitation, College of Health Sciences, Nnamdi Azikiwe University, Nnewi Campus, Nnewi, Anambra State Nigeria

**Keywords:** Cross-cultural adaptation, Validation, Modified falls efficacy scale, Igbo

## Abstract

**Background:**

Fear of falling (FOF) is a very pervasive problem among older adults. Consequently, many scales have been developed for its assessment. The Modified Falls Efficacy Scale (MFES) is one of the most popular FOF scales. The MFES was originally developed for use in developed countries, and thus may not be entirely suitable for use in developing countries due to cultural and environmental differences between the two country categories. This study was therefore designed to cross-culturally adapt and validate the MFES to Igbo culture and environment among community-dwelling older adults in Nnewi community using established guidelines.

**Methods:**

The original English version of the MFES (E-MFES) was translated, synthesized, back-translated, subjected to expert panel review, and pretested before producing the final Igbo version of the MFES (I-MFES). The I-MFES and the Short Falls Efficacy Scale International were randomly administered to consecutively recruited 109 consenting older adult residents of Nnewi (43.1% males; mean age = 74.45 ± 8.78 years). Convergent and structural validities and internal consistency of the I-MFES were assessed at 0.05 level of significance.

**Results:**

All the 14 items on the E-MFES were retained on the I-MFES. The I-MFES exhibited the same structure as the E-MFES. The correlation between the total scores on the I-MFES and the Short Falls Efficacy Scale International was excellent (rho = − 0.93) indicating evidence of convergent validity of the I-MFES. The Cronbach’s alpha value of the I-MFES was 0.97 showing evidence of excellent internal consistency of the items on the I-MFES.

**Conclusion:**

This study provides evidence of some aspects of validity and reliability of the I-MFES.

## Background

Ageing is usually associated with a lot of health challenges [1–3]. Falls and fear of falling are some of such challenges that may have serious pervasive effects among older adults [4, 5]. Fall, defined as an event in which the individual inadvertently comes to rest on the ground or lower level against his will, is a public health issue among older adults [6, 7]. It is generally estimated that one in three older adults suffers a fall yearly [6] and prevalence rates of 19 to 27.8% had been reported among Nigerian older adults [8, 9]. One in five falls among older adults causes serious injuries (such as fractures or head injuries), thereby making falls one of the most common causes of hospitalization and death in this group. This implies that falls have an excessive bearing on health care expenses worldwide [10, 11]. Apart from the physical and financial burden, fall is also reported to have significant psychological and social consequences, with many older adults who had experienced a fall becoming apprehensive or fearful of fall [4].

Fear of falling, described as the ongoing concern about falling that ultimately limits the performance of daily activities, is highly prevalent among community-dwelling older adults [4], and tends to be even higher among those that have experienced fall or that are under institutionalized care. The prevalence of fear of falling varies across communities and societies with rates of 34.4, 35, 43.3 and 95.2% having been reported among populations in Nigeria, Europe, Japan and Brazil respectively [9, 12–14]. An FOF prevalence of 81% had also been reported in a Nigerian stroke sample [15] suggesting that along with age, the presence of any co-morbidity that is likely to interfere with balance may heighten the likelihood of FOF. Fear of falling (FOF) is a common consequence of falls that can lead to a lot of physical and psychological problems among older adults [16]. It has been associated with limitation and/or reduction in the performance of activities of daily living, mobility, physical capability, mental health, balancing activities and quality of life, and increased institutionalization [1, 9, 10, 17]. Fear of falling is suggested to be a potential health problem of equal importance to a fall or may even be a more pervasive and serious problem than actual falls in older adults [18, 19]. Consequently, FOF is routinely investigated in older adults and several instruments have been developed for its measurement.

The Falls Efficacy Scale (FES) is one of the most common instruments for assessing fear of falling [20]. In a bid to improve the instrument, four more items were added to the FES to produce a 14-item valid and reliable Modified Falls Efficacy Scale (MFES) [21] which had since gained popularity in literature. The MFES, like other FOF measures, was originally produced to assess FOF in developed countries. As a result, it may not be entirely suitable for use in developing countries due to cultural and environmental differences between the two categories of countries [22]. According to Beaton et al. [22], for an instrument to be used in a new language, setting, culture and environment, it must be cross-culturally validated in order to ensure semantic, idiomatic, conceptual and experiential equivalences between the original and the target populations.

When faced with the problem of unavailability of environment- and culturally-specific outcome measures for assessing a particular construct in a particular setting, stakeholders are usually faced with two options: development of an entirely new scale or cross-culturally adapting the existing scale to suit the new setting. It is usually better to cross-culturally adapt an existing scale than developing a new instrument as cross-cultural adaption is more economical and allows for comparison across populations and locations [22, 23]. Cross-cultural adaption usually includes initial translation, synthesis, back translation, expert committee review, pilot testing and psychometric evaluation [22, 24]. With the Nigerian adult literacy in English language standing at 42.1% [25], many Nigerian older adults will not be able to complete the original English versions of the MFES and other FOF scales. This may introduce bias in assessing FOF among Nigerian older adults as no Nigerian-adapted FOF scale is readily available. Mere translating the instruments to this group of participants by different assessors will equally introduce some biases as the translations are not validated and may vary markedly with individuals. This study was therefore designed to cross-culturally adapt and validate the MFES among Igbo older adults in Nnewi North Local Government Area.

## Methods

### Design

This is a validation study that employed the guidelines for cross-cultural adaptation developed by Beaton et al. [22] for the American Association of Orthopaedic Surgeons. The Ethical Review Committee of Nnamdi Azikiwe University Teaching Hospital gave approval for this study before commencement of data collection. Written informed consent was obtained from each consecutively recruited Igbo community-dwelling older adult (65 years and above) residents of Nnewi, a semi-urban commercial and agrarian community in Anambra State of South-east Nigeria. Igbo is one of the three major native languages in Nigeria (forming about 18% of the whole Nigerian population) and a minor language in Equatorial Guinea, with over 24 million speakers [26–28]. Participants for this study: could understand both English and Igbo languages; could walk independently (with or without assisted devices); were either fallers or non-fallers; were well-oriented in time, place and person; and were not selected based on medication usage. Older adults with dementia were excluded from the study. These criteria were for both the pretesting and validation of the questionnaire.

### Instruments

#### The modified falls efficacy scale (MFES)

This 14-item activity questionnaire is an expanded version of the original 10-item activity Falls Efficacy Scale. The MFES includes outdoor activities (transportation, crossing roads and light gardening and hanging out the washing) which the FES does not cover. Each item is scored on the 10-point visual analogue scale: 0 = not confident or not sure at all, 5 = fairly confident or fairly sure, and 10 = completely confident or completely sure. The total score is the average of all the item scores. Hence, the total score ranges from zero to ten. Higher scores reflect more confidence and less fear of falling while lower scores reflect less confidence and more fear of falling. Participants are classified as either fearful (MFES score < 8) or not fearful (MFES score ≥ 8) based on an earlier proposition [21]. A high reliability was reported in older sample of fallers and non-fallers. The MFES demonstrated high internal consistency (Cronbach’s alpha = 0.95), high retest reliability (ICC = 0.93), and less skewness than the original FES [21].

#### The short falls efficacy scale international (FES-I)

This was used to assess the construct (convergent) validity of the MFES. It is a 7-item tool that is one of the most commonly used scales for assessing fear of falling [20]. It assesses concerns about falls in seven activities: dressing, bathing, getting in or out of a chair, ascending and descending a staircase, ascending and descending a slope, reaching out for something overhead or on the ground, and going out for social events. Each item has a four-point Likert format response options (ranging from ‘not at all concerned’ to ‘very concerned’) that are rated from 1 to 4. Total score is the summation of the individual item scores, and is ranged from 7 (no concern about falling) to 28 (severe concern about falling). The scoring is interpreted thus: low concern (7–8); moderate concern (9–13); and high concern (14–28). The short FES-I displayed excellent internal consistency (Cronbach’s alpha = 0.92), test-retest reliability (ICC = 0.83) and construct validity (*r* = 0.97) [29].

#### Cross-cultural adaptation and validation of the MFES

The American Association of Orthopedic Surgeons’ guidelines for cross-cultural adaptation and validation of pen and paper instruments as developed by Beaton et al. [22] was followed in the present study. It is categorized into three stages: translation, adaptation and validation.

#### Phase one: translation

This stage involved forward translation of the original English language version of the MFES (E-MFES) into Igbo language. A physiotherapist and a linguist who were bilingual and had Igbo language as their mother tongue independently translated the questionnaire. Unlike the second translator, the first translator had medical background and was aware of the construct (fear of falling) that the E-MFES is assessing. The second translator provided translation equivalent to that of the general population by highlighting ambiguous meanings in the original questionnaire. Two translations were thus produced (T1 and T2). The two translators came together and harmonized the two translations, and subsequently synthesized a common version (T12). The synthesized version (T12) was translated back into English language by two other different translators who did not know about the E-MFES. These back-translators were Igbo physiotherapy lecturers who were experienced in the process of cross-cultural adaptation. Their mother tongue was Igbo language as against English language recommended by Beaton et al. [22]. This is as a result of lack of bilingual translators whose mother tongue was English language. This kind of improvisation has been used often in literature when it is difficult to get a native speaker of the original language who is also fluent in the target language [30–32]. Two back translated English versions (BT1 and BT2) were thus produced. This procedure helped in identifying errors in the original questionnaire and also to make sure that the translated version reflected the same item content as the original version.

#### Phase two: adaptation

The E-MFES, the two forward translations (T1 and T2), the synthesis (T12) and the backward translations (BT1 and BT2) were reviewed by a panel of experts whose aim was to cross-culturally adapt the synthesis (T12) to Igbo language and culture. The expert panel included all the four translators, and five physiotherapy researchers who were experienced in the process of cross-cultural adaptation, and a lay person. The expert panel checked the relevance, comprehensiveness and comprehensibility of the instructions, items and response options. All the translations were considered while ensuring conceptual, semantic, idiomatic and experiential equivalence between the Igbo and the English versions of the scale. Discrepancies in the different translations of the questionnaire were resolved through general consensus. A pre-final Igbo version of the MFES was thus produced. The pre-final Igbo version was pretested among 30 older adults (70% female; mean age 72.7 **±** 7.63 years) who were consecutively recruited from conveniently selected Okofia community of Nnewi North Local Government Area. The participants had at least secondary education. They were equally taken through the process of cognitive debriefing interview. The participants were queried on: the clarity and ease of understanding of the items and the response options; if each activity is practiced in Igbo culture; and if they believed that the scale covered all the necessary aspects of lives as it concerns fear of falling. The participants were expected to answer ‘YES’ or ‘NO’ for each question on each item and response options. The information from the pretesting was considered by the expert panel in a second meeting. Any item or response option with less than 80% ‘YES’ response was supposed to be amended. However, all the items and response options made at least 80% ‘YES’. As a result, no further modification was made on the questionnaire by the expert panel. The final Igbo version of the MFES (I-MFES) was thus produced.

#### Phase three: validation

The I-MFES and the FES-I were either self-administered or researcher-administered to the participants depending on each participant’s preference. The order of the administration of the two questionnaires was randomized using simple randomization method. All participants who picked the letter “I” responded to the I-MFES first while all those that picked the letter “F” responded to the FES-I first. According to COSMIN (Consensus-based Standards for the selection of health Measurement INstruments) a sample size of ≥100 or seven times number of items on a scale is very good for ascertaining structural validity, internal consistency and construct validity of the scale [33]. A sample size of 109 was therefore used in the present study. Participants were consecutively recruited from conveniently selected communities in Nnewi North Local Government Area.

### Analysis of data

Data was analyzed using the Statistical Package for Social Sciences (SPSS) (version 21). The demographic and clinical variables as well as the scores from the questionnaires (the I-MFES and the FES-I) were summarized using frequency counts, percentages, range, mean and standard deviation. The participants’ scores on the I-MFES were tested for normality using the Kolmogorov-Smirnov’s test. The Spearman’s rank order correlation test was used to estimate the level of correlation between participants’ scores on the I-MFES and the FES-I (in order to provide evidence of convergent validity of the I-MFES). Scatter plots were used to pictorially depict this correlation. The Cronbach’s alpha was used to determine the internal consistency of the items on the I-MFES. The standard error of mean (SEM) and the minimal detectable differences (MDD) of the item and total scores on the I-MFES were also calculated. The MDD was calculated using the formula MDD = 1.96 x SEM x √2 [27]. The principal component analysis (PCA) was used to determine the structural validity of the I-MFES. For data to be suitable for PCA: all the correlation matrix coefficients between each item pair must be above 0.3; the Kaiser-Meyer-Olkin measure of sampling adequacy must exceed the recommended value of 0.6; the Bartlett’s test of sphericity must reach statistical significance; and the communalities must all be above 0.3 [34–37]. During PCA, only factors with eigen value of greater than one are usually retained [38]. Catell’s Scree test [39] was also used to decide on the number of factors to retain for further investigation. Finally, the Monte-Carlo parallel analysis was used to decide the number of components to retain by considering the components with eigen values exceeding the criterion value for a randomly generated data matrix of the same size (14 variables × 109 respondents). The alpha level was set at 0.05.

## Results

### Cross-cultural adaptation of the MFES into Igbo

Translation of most items on the E-MFES revealed no controversy. All the 14 items on the original MFES were retained but item 8 was slightly modified. The terms “cabinets” and “closets” in item 8 had no Igbo equivalent terms. The expert review committee agreed that the Igbo equivalent of “cupboard” could give the most similar meaning. All the participants who were involved in the pretesting process reported clarity of language and ease of understanding of all the items during the cognitive debriefing interview. The participants also agreed that the term used to replace “cabinets” and “closets” was suitable and Igbo friendly description for the terms. At the second expert panel meeting, the consensus was that the cross-cultural adaptation of the MFES into Igbo was good and that participants’ responses at pretest justified the term adapted for “cabinets” and “closets”. No further modification was made.

### Validation of the I-MFES

A total of 109 older adults (43.1% males) with mean age of 74.45 ± 8.78 years participated in the psychometric testing of the I-MFES. 14.7% of the participants had lost their spouses, and 67.9% had attained at least secondary level of education. Majority (82.6%) of the participants were still occupationally active with farming (37.6%) being the most predominant occupation (Table 1).

**Table 1:** Socio-demographic profiles of the participants

Variable	Class	Frequency	Percentage (%)
Gender	Male	47	43.1
	Female	62	56.9
Marital status	Single	1	0.9
	Married	92	84.4
	Widowed	16	14.7
Highest educational attainment	Primary	35	32.1
	Secondary	62	56.9
	Tertiary	12	11.0
Occupational status	Unemployed	6	5.5
	Retiree	13	11.9
	Farming	41	37.6
	Trading	26	23.9
	Civil/Public service	18	16.5
	Artisan	5	4.6

### Validities and reliabilities of the Igbo version of the I-MFES

All the I-MFES scores (both item and total) fell within the range of being “fearful”. The participants scored highest and lowest in items 6 (answering the door or telephone) and 12 (crossing the road) respectively on the I-MFES. The participants’ scores on the I-MFES were not normally distributed (as their *p*-values were less than 0.05) thus necessitating the use of nonparametric statistics on the scores.

**Fig. 1:**
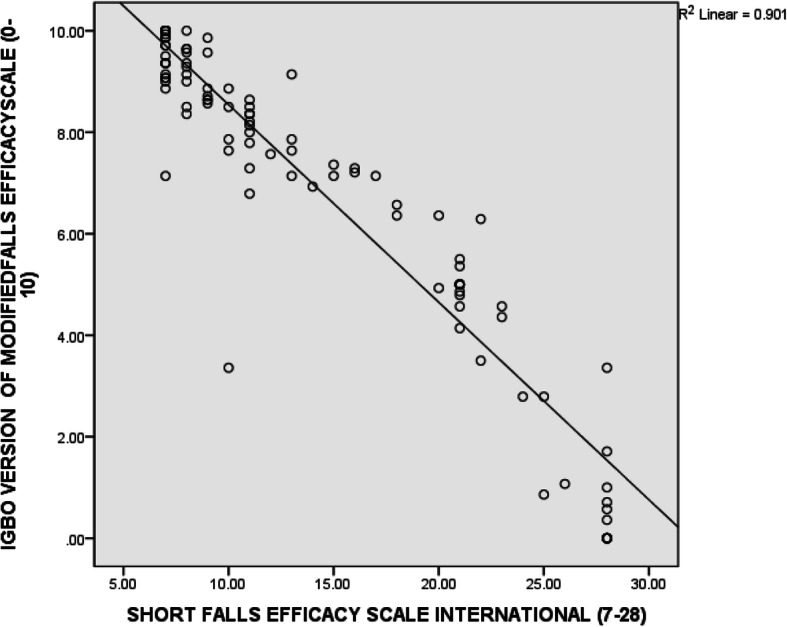
Scatter diagram for the correlation between total scores on the Igbo version of the Modified Falls Efficacy Scale and the Short Falls Efficacy Scale International

The convergent validity coefficient (rho = − 0.93) of the I-MFES estimated by correlating the total scores on the I-MFES and the FES-I was excellent, indicating that the two instruments measure the same construct (fear of falling). The correlation between the total scores on the I-MFES and the FES-I is pictorially represented on a scatter plot (Fig. 1). The minimal detectable difference (MDD) of the item and total scores on the I-MFES ranged from 0.79 to 0.89, and could give insight into the responsiveness of the item and total scores on the scale. Items 3 and 7 had the highest and lowest SEM and MDD scores respectively (Table 2). Internal consistency coefficient (alpha = 0.97) of the items on the I-MFES estimated by means of Cronbach’s alpha using the split half method was excellent.

**Table 2:** Standard error of mean and minimal detectable difference of the item and total scores on the Igbo version of the modified falls efficacy scale

I-MFES scores	SEM	MDD
Item 1	0.30	0.84
Item 2	0.30	0.82
Item 3	0.32	0.89
Item 4	0.31	0.85
Item 5	0.30	0.84
Item 6	0.29	0.80
Item 7	0.29	0.79
Item 8	0.32	0.88
Item 9	0.32	0.87
Item 10	0.30	0.84
Item 11	0.32	0.87
Item 12	0.32	0.88
Item 13	0.31	0.86
Item 14	0.31	0.87
Total score	0.29	0.80

*I-MFES* Igbo version of the Modified Falls Efficacy Scale, *SEM* Standard error of mean, *MDD* Minimal detectable difference

### Structural validity of the Igbo version of the I-MFES

**Fig. 2:**
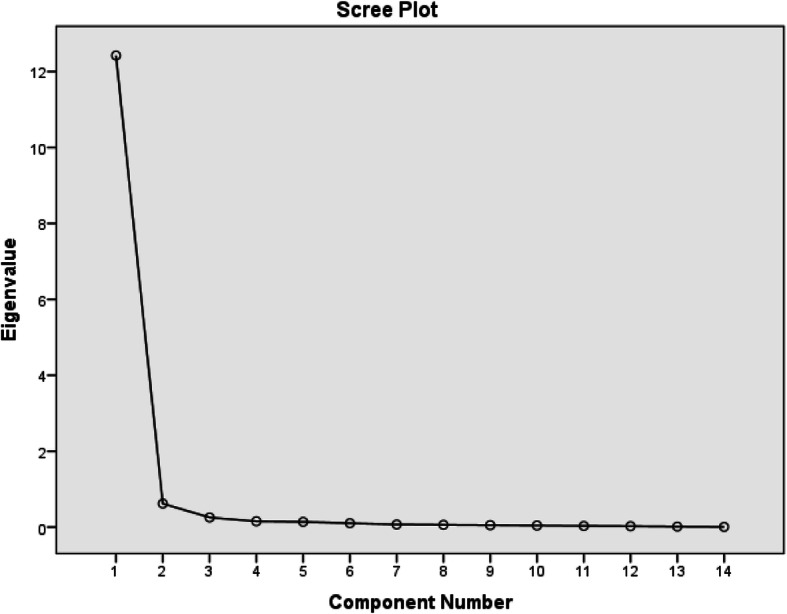
Scree plot of the components on the Igbo version of the Modified Falls Efficacy Scale

The data was fit for factorial analysis. Inspection of the correlation matrix revealed that all the coefficients were above 0.3, which suggested reasonable factorability. The Kaiser-Meyer-Olkin measure of sampling adequacy was 0.94 exceeding the recommended value of 0.6, and Barlett’s test of sphericity reached statistical significance (X^2^(91) =3405.93; *p* = 0.0001) supporting the factorability of the correlation matrix. Finally, the communalities were all above 0.3 (Table 3), thus confirming that each item shared some common variances with other items. Given these overall indicators, factor analysis was conducted with all the 14 items of the I-MFES. The PCA revealed the presence of only one component with the initial eigen value exceeding 1, accounting for 88.73% of the variances (Table 4). Scree plot also confirmed the presence of one component by showing a clear reflection after the first factor (Fig. 2). This structure is similar to that of the original English version of the MFES (E-MFES) which has only a total but no domain scores.

**Table 3:** Communalities of the items on the I-MFES

Items	Initial	Extraction
I_MFES1	1.00	0.91
I_MFES2	1.00	0.88
I_MFES3	1.00	0.86
I_MFES4	1.00	0.92
I_MFES5	1.00	0.92
I_MFES6	1.00	0.90
I_MFES7	1.00	0.88
I_MFES8	1.00	0.89
I_MFES9	1.00	0.90
I_MFES10	1.00	0.91
I_MFES11	1.00	0.83
I_MFES12	1.00	0.79
I_MFES13	1.00	0.93
I_MFES14	1.00	0.91

*Extraction Method* Principal Component Analysis, *I-MFES* Igbo version of the MFES

**Table 4:** Factor analysis and Monte Carlo principal component analysis for parallel analysis of Igbo version of the MFES

Factor	Random Eigen values	Decision	%variance	%cumulative
Factor1	12.422	Accept	88.73	88.73
Factor 2	0.620	Reject	4.43	93.16
Factor 3	0.26	Reject	1.82	94.98
Factor 4	0.154	Reject	1.10	96.08
Factor 5	0.140	Reject	1.00	97.08
Factor 6	0.104	Reject	0.74	97.82
Factor 7	0.071	Reject	0.51	98.32
Factor 8	0.061	Reject	0.44	98.76
Factor 9	0.051	Reject	0.34	99.12
Factor 10	0.043	Reject	0.31	99.43
Factor 11	0.031	Reject	0.22	99.65
Factor 12	0.026	Reject	0.19	99.84
Factor 13	0.015	Reject	0.11	99.95
F Factor14	0.007	Reject	0.05	100.00

## Discussion

The present study was designed to cross-culturally adapt and validate the Igbo version of the Modified Falls Efficacy Scale among Igbo older adults in Nnewi North Local Government Area. In order to ensure accuracy and reduction of bias, the procedure for this study followed the established guidelines for cross-cultural adaptation and validation of pen and paper instruments [22]. During the process of translating the original English version of the MFES into Igbo version (I-MFES), minimal difficulty was encountered in achieving an acceptable translation. All the items on the MFES were considered by the expert panel to be relevant for measuring level of fear of fall among Igbo speaking older adults. However, few modifications were made in order to ensure semantic, experiential and conceptual equivalence of the terms and examples in Igbo environment. The terms “cabinets” and “closets” in item 8 had no Igbo equivalent terms and were replaced with an Igbo equivalence of “cupboard”. Beaton et al. [22] suggested that during cultural adaptation of pen and paper instrument, equivalence of the old instrument in the new culture should be ensured. After this adaptation, the I-MFES was then pretested on 30 older adults who were also engaged in cognitive debriefing interview. There was a general consensus of clarity and ease of understanding of all the 14 items among the participants who engaged in cognitive debriefing interview. Hence all items were adopted as suggested in literature [22]. All these suggest that the translated version is a good equivalence of the original instrument.

Consequently, the I-MFES may be used in place of the E-MFES among Igbo-speaking older adults, irrespective of where they might be found. Generally, the Igbos dwell in the South-east Nigeria but have migrated to many other parts of the world. However, they have always maintained a core linguistic pattern anywhere they find themselves. Even though the Igbo language consists of various dialects, it maintains a central cultural and linguistic pattern which is well understood by most Igbo language speakers. The MFES has been translated into various languages (e.g. German [29], Dutch [20], and Serbian [40]. As directed in literature, these translations are used in place of the original English version in these places and anywhere the speakers of these languages are found. In the same vein, the I-MFES can be used in place of the E-MFES on any Igbo speaker that is most comfortable with Igbo Language irrespective of where they reside.

The correlation coefficient of the relationship between sum scores on the I-MFES and the FES-I demonstrates that I-MFES has excellent convergent validity. This implies that the two instruments (the I-MFES and the FES-I) assess the same construct which is FOF. The I-MFES exhibited excellent internal consistency shown by a Cronbach’s alpha value of 0.97 which falls within the acceptable range. The Cronbach’s alpha value of 0.97 is similar to previously reported values in Serbian (0.98) [40], French (0.94) [41] and Chinese (0.90) [42] versions of the MFES. This finding indicates that the items on the I-MFES are homogenous and internally consistent and that each is assessing different aspects of the construct (FOF) being evaluated. The MDD of the I-MFES reported in this study will be useful in the future in knowing when there is a significant change in FOF of older adults, for instance following an intervention.

Factor analysis is intimately involved with the question of validity, and it is the center of the measurement of psychological constructs [31, 43]. It provides a diagnostic tool to evaluate whether the collected data are in line with the theoretically expected pattern, or structure of the target construct and thereby determine if the measures used have indeed measured what they are purported to measure (construct validity). Principal component analysis was chosen as against the exploratory factor analysis because of the fact that the scale has already been established on an existing theory by the original authors of the English version [43]. The data met all the criteria for PCA. The Kaiser-Meyer-Olkin value was within the acceptable limit of greater than 0.6. The KMO values below 0.6 should have led to either collection of more data or a rethink of which variable to include. The Barlett’s test of sphericity also revealed that significant correlation exists in the random matrix. These findings suggest that the I-MFES is a good measure of FOF, and that it measures FOF just as good as the E-MFES in the sampled population. PCA revealed that the I-MFES has only one component similar to what obtained in the original English version. This shows that the I-MFES has only item and total scores without a domain score.

The study is not without limitations. Participants of the present study were recruited from a single community rather than from diverse communities all over Igbo land. We however reasoned that the use of the central Igbo language commonly understood by all will play a big role in minimizing the effect of the recruitment bias. Furthermore, native speakers of English language who were also fluent in Igbo language could not be found to back-translate the harmonized Igbo version of the MFES into English language. The services of lecturers in Physiotherapy who were very fluent in both languages, and were equally very experienced with cross-cultural adaptation were employed. It is believed that their experience in cross-cultural validation would have ensured qualitative translation process.

## Conclusion

The translated Igbo version of the Modified Falls Efficacy Scale (I-MFES) is an internally consistent tool that has demonstrated good convergent and structural validity and thus can be used as an outcome measure for Igbo monolingual older adults. It is recommended that the I-MFES be translated and validated into other major Nigerian (Hausa and Yoruba) and international languages, as availability of this instrument in different languages may help enhance its utility across various cultures. Future studies can explore other psychometric properties of the I-MFES like the intra-rater and inter-rater reliabilities, responsiveness, predictive and discriminant validities.

## Data Availability

The dataset used and/or analysed during the current study are available from the corresponding author on reasonable request.

## Abbreviations

FOF: Fear of falling
MFES: Modified Falls Efficacy Scale
E-MFES: Original English version of the Modified Falls Efficacy Scale
I-MFES: Igbo version of the Modified Falls Efficacy Scale
WHO: World Health Organisation
FES-I: Falls Efficacy Scale International
T1: First forward translation of the Modified Falls Efficacy Scale
T2: Second forward translation of the Modified Falls Efficacy Scale
T12: Synthesized Igbo version of the Modified Falls Efficacy Scale
BT1: First backward translation of the Modified Falls Efficacy Scale
BT2: Second backward translation of the Modified Falls Efficacy Scale
SEM: Standard error of mean
MDD: Minimal detectable differences
PCA: Principal component analysis
